# The Effect of Lavender Aromatherapy on Autonomic Nervous System in Midlife Women with Insomnia

**DOI:** 10.1155/2012/740813

**Published:** 2011-08-18

**Authors:** Li-Wei Chien, Su Li Cheng, Chi Feng Liu

**Affiliations:** ^1^Department of Obstetrics and Gynecology, Taipei Medical University Hospital, Taipei, Taiwan; ^2^Department of Obstetrics and Gynecology, School of Medicine, College of Medicine, Taipei Medical University, Taipei, Taiwan; ^3^National Taipei University of Nursing and Health Sciences, Taipei, Taiwan; ^4^Graduate Institute of Integration of Traditional Chinese Medicine with Western Nursing, National Taipei University of Nursing and Health Sciences, No. 365 Ming-De Road, Beitou, Taipei 11211, Taiwan

## Abstract

The objective of this study is to determine the effects of 12 weeks of lavender aromatherapy on self-reported sleep and heart rate variability (HRV) in the midlife women with insomnia. Sixty-seven women aged 45–55 years, with a CPSQI (Chinese version of Pittsburgh Sleep Quality Index) greater than 5, were recruited from communities in Taiwan. The experimental group (*n* = 34) received lavender inhalation, 20 min each time, twice per week, for 12 weeks, with a total of 24 times. The control group (*n* = 33) received health education program for sleep hygiene with no intervention. The study of HRV was analyzed by time- and frequency-domain methods. Significant decrease in mean heart rate (HR) and increases in SDNN (standard deviation of the normal-to-normal (NN) intervals), RMSDD (square root of the mean squared differences of successive NN intervals), and HF (high frequency) of spectral powers analysis after lavender inhalation were observed in the 4th and 12th weeks of aromatherapy. The total CPSQI score of study subjects was significantly decreased in the experimental group (*P* < 0.001), while no significant difference was observed across the same time period (*P* = 0.776) in the control group. Resting HR and HRV measurements at baseline 1 month and 3 months after allocation showed no significant difference between the experimental and control groups. The study demonstrated that lavender inhalation may have a persistent short-term effect on HRV with an increase in parasympathetic modulation. Women receiving aromatherapy experienced a significant improvement in sleep quality after intervention. However, lavender aromatherapy does not appear to confer benefit on HRV in the long-term followup.

## 1. Introduction

Subjective reports of difficult sleeping are prevalent in middle-aged women undergoing the menopausal transition. Sleep problems are reported in 39–47% of perimenopausal women, and 35–60% of postmenopausal women from the data presented at the NIH State-of-the-Science Conference on Management of Menopause-Related Symptoms [1]. In Taiwan, 46% of the middle-aged women felt dissatisfied with their sleep in a community-based study [2]. The incidence is consistent with prior studies in other patient populations, and the underlying mechanisms of poor sleep quality among these women were related to anxiety [2]. Sleep disturbances have been considered as a natural part of the perimenopausal process in Taiwan [3–5]. Perimenopausal women generally believe that their sleep problems will subside as they pass through menopause transition period. Therefore, they tended to seek self-help strategies or complementary and alternative medicine (CAM) before resorting to western medicine [3–5].

Among the CAM, aromatherapy has been widely used to modify mood and sleep [6]. Aroma inhalation of lavender, chamomile, and ylang-ylang induces a state of mind conducive to sleep. Short-term inhalation of lavender oil has been used as a sleep aid [6]. In a single-blinded, randomized pilot study, Lewith and colleagues investigated the efficacy of lavender essential oil on insomnia in 10 volunteers and discovered that the lavender oil created an improvement in the sleeping quality more effectively than sweet almond oil in the control group [7]. Using a placebo, a control, and a treatment, Goel and colleagues found that lavender was able to increase the percentage of deep or slow-wave sleep in both men and women [8]. Lavender also was found to increase stage 2 (light) sleep and decrease rapid eye movement (REM) sleep and the amount of time to wake up after first falling asleep (waking up after sleep onset latency) in women, with opposite effects in men. It was concluded that lavender may provide a mild sedation to promote sleep [7, 8]. However, there is insufficient evidence from the current investigations to prove direct hypnotic effect of lavender oil in treating insomnia [9, 10].

On the other hand, there are evidences for the effectiveness of lavender in the treatment of anxiety in humans [11]. In a single-blinded, randomized pilot study, Lehrner and colleagues found lavender odor exposure to be effective in relieving anxious mood in dental patients who underwent a stressful procedure [12]. Kuroda and colleagues examined the sedative effects of (R)-(−)-linalool, the main component of both jasmine tea and lavender, on moods and autonomic nerve activity (ANS) [13]. They found that it produced calm and vigorous mood state and elicited a significant decrease in heart rate and an increase in HF (high-frequency) component when compared with the controls [13]. To examine whether the odor is required for lavender to exert its anxiolytic effects, Bradley and colleagues tested the efficacy of lavender administered orally. They showed that lavender capsules have beneficial effects in relieving mild anxiety with significantly increased heart rate variability (HRV) in females but not in males [14]. These studies demonstrated that lavender's anxiolytic and stress-relieving effects might manifest as modulation of HRV.

Most of the published data on the physiological effects of lavender were tested only in a short duration [11–14]. Therefore, the purpose of this study was to evaluate the efficacy of lavender aromatherapy on HRV before and after 12 weeks of treatment in midlife women with insomnia. We hypothesized that lavender aromatherapy would affect both autonomic modulations and sleep quality after a follow-up period of 3 months.

## 2. Methods

### 2.1. Subjects

This was a prospective study conducted from November 2007 to June 2008. All study procedures were approved by the committee at the National Taipei College of Nursing. Volunteers were recruited voluntarily from communities in Taipei under a healthcare program regarding sleep hygiene. All participants met the following criteria: (1) age between 45 and 55 years; (2) conscious clear and available verbal communication; (3) no symptoms of dysosmia; (4) currently not receiving any hormone-replacement therapy. All potential subjects were screened for sleep quality with a Chinese version of the Pittsburgh Sleep Quality Index (CPSQI). The CPSQI has been demonstrated to be a psychometrically sound measure of sleep quality with acceptable test-retest reliability over a 14- to 21-day interval with a coefficient of 0.85 for all subjects and 0.77 for primary insomniacs [15]. A CPSQI global score of greater than 5 yielded a sensitivity of 98% and a specificity of 55% as a marker for poor sleep in primary insomniacs versus controls [15]. A total of 67 midlife women who met the above criteria with a CPSQI greater than 5 were recruited. They were randomly assigned to the experimental group (*n* = 34) and the control group (*n* = 33) based on register code entry by a computer. The control group did not receive any intervention. Written consent was obtained before their participation in the study.

### 2.2. Procedures

**Figure 1:**
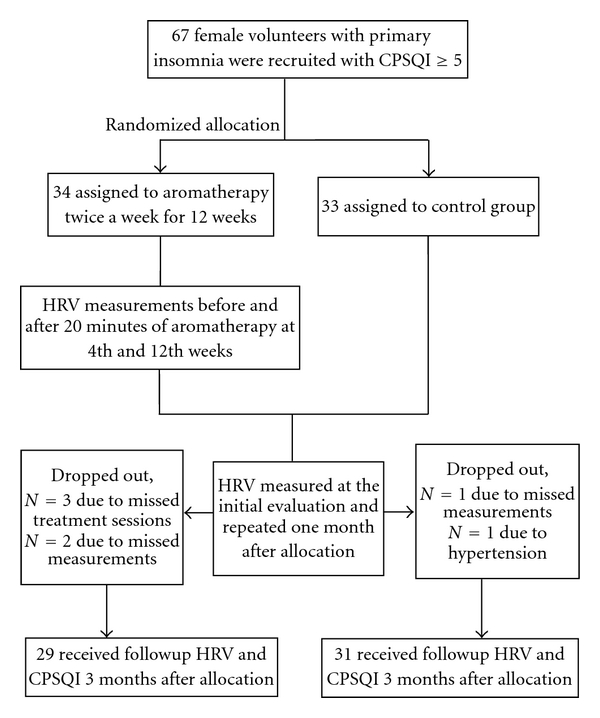
Flowchart of the distribution of the cohort study.

 All studies were performed in the evening after a regular work between 17:00 and 23:00 hours. The participants were required to abstain from drinking caffeinated or alcohol-containing beverage at least 3 h prior to testing. All experiments were conducted in a bright and quiet room; the ambient temperature was kept at 22–25°C. Subjects were in a sitting position, providing easy access to attach the electrodes, and were encouraged to relax. After relaxing in a comfortable chair with arm supports for 10 min, aromatherapy was carried out by inhalation after instilling 0.25 c.c. essential lavender oil (Australian Certified Organic Pty Ltd, Brisbane, Australia) and 50 c.c. of water into an ultrasonic ionizer aromatherapy diffuser (Heavenly Scent, YHL International Co., Ltd, Taipei, Taiwan). They were kept at a distance of 10–15 cm away from the diffuser, and the inhalation lasted for 20 min each time. Participants in the aromatherapy arm received a 12-week course of twice a week with a total of 24 times. Control studies without aromatherapy were performed in an environment as described above in the experimental group. A flow diagram for collecting data is shown in Figure 1.

### 2.3. Heart Rate Variability (HRV) Analysis

The subjects were instructed to avoid strenuous physical activity and alcohol and coffee intake 24 h before the measurement. Before recording the HRV, the participants practiced controlling their breathing rhythm to follow the metronome. During the HRV recording, participants were resting in a sitting position and breathing in a controlled rate of 12–15 breaths per min. After 10 min of rest in a sitting position, an initial measurement of HRV was performed. HRV was recorded in the sitting position using the heart rate variability analyzer (SA-3000P, Medicore Co., Ltd, Seoul, Korea). The sensor of HRV was clipped on the index finger (second finger) of the left hand. The subjects were asked to keep silence and stay inactive during the measurement. HRV measurement was performed at the baseline measurement, in the 4th and after the 12th weeks of follow-up period in both experimental and control groups. In the experimental group, HRV measurements were performed before and 10 min after aromatherapy in the 4th and 12th weeks of the treatment.

HRV analysis was performed according to the guidelines of the Task Force of the European Society of Cardiology [16]. Time domain measures quantify the mean heart rate (HR) and SDNN (ms), that is, the standard deviation of the normal-to-normal (NN) intervals: RMSSD (ms), that is, the square root of the mean squared differences of successive NN intervals. These measures are considered to be indices of cardiac parasympathetic activity [17, 18]. Frequency domain measures are derived from spectral analysis of *N*-*N* intervals and may be expressed in either absolute or normalized terms. Absolute terms including the total power (TP), the very-low-frequency power (VLF: 0.00–0.04 Hz), the low-frequency power (LF: 0.04–0.015 Hz), and the high-frequency power (HF: 0.15–0.40 Hz) were calculated using a 3-minute window. Normalized unit spectral power measures (HFnu, LFnu) are derived from their absolute power equivalents (HF, LF) over a normalizing denominator of the total power (from which VLF has been subtracted) and multiplying by 100. Overall, VLF, HF, and HFnu represent parasympathetic activity; LF and LFnu represent both sympathetic and parasympathetic activity. Finally, the LF/HF or LFnu/HFnu ratio is considered to reflect sympathovagal balance [17, 18].

### 2.4. Statistical Analysis

Categorical variables were compared by Chi-square/Fisher's exact test and nonparametric data on HRV parameters at baseline, 1 month after treatment, and after 3 months between the two groups they were compared using the Mann-Whitney *U*-test. In addition, Wilcoxon signed-rank tests were employed to analyze the HRV parameters before and after treatment in the experimental group. Categorical data were represented by number (%) and nonparametric data presented as median (interquartile range). All statistical assessments were two-sided and evaluated at the 0.05 level of significant difference. Statistical analyses were performed using SPSS 15.0 statistics software (SPSS Inc., Chicago, Ill, USA).

## 3. Results

Among the randomized participants, 7 (10.4%) did not complete the study. In the experimental group, 29 of 34 patients (85.3%) completed the study while 5 withdrew early, and in the control group, 31 of 33 patients (93.9%) completed the study while 2 withdrew from it. The demographics and characteristics of study subjects are shown in Table 1. There were no statistically significant differences in demographics and characteristics between the two groups (*P* > 0.05).  Table 2 represents the comparison of HRV parameters in the 4th and 12th weeks of treatment in the experimental group. In the 4th week, there were significant differences in mean HR, SDNN, RMSSD, and HF before and after aromatherapy in the experimental group (*P* < 0.05). In the 12th week, there were significantly declined mean HR and increased SDNN, RMSSD, and HF after lavender inhalation (*P* < 0.05).

**Figure 2:**
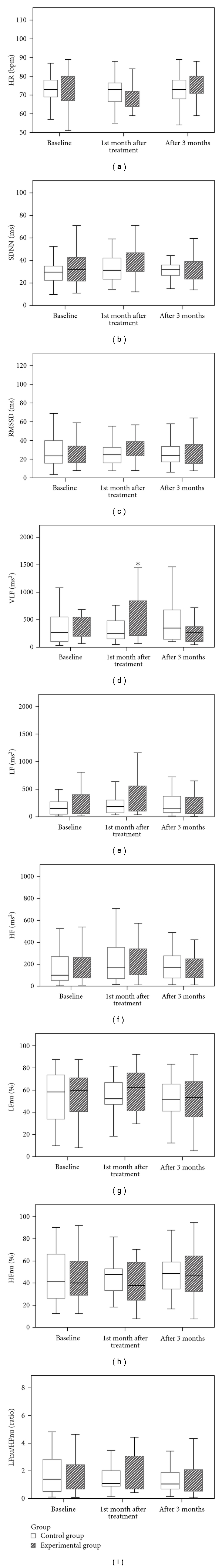
Comparison of HRV parameters during the study period. *indicates a significant difference in VLF between the two groups at the first month after treatment using Mann-Whitney *U*-test (*P* < 0.05).

The differences in the HRV parameters between the two groups at baseline 1 month and 3 months after allocation are shown in Figure 2, indicating a significant difference in VLF at the first month after treatment between the experimental and control groups (*P* = 0.040). The median VLF level in experimental group was significantly higher than those in the control group. All the other HRV measurements, however, did not show significant difference between the lavender aromatherapy and control group.

**Figure 3:**
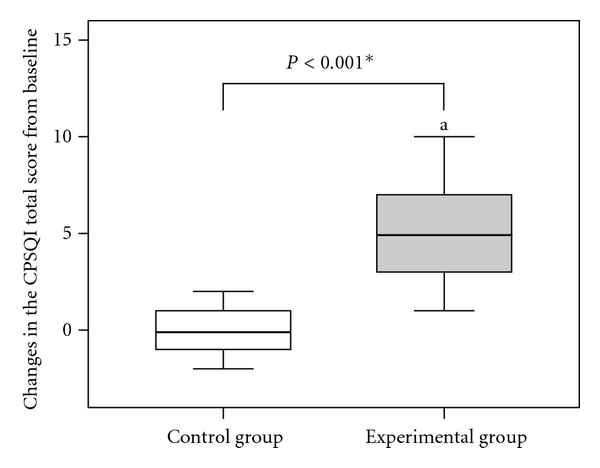
Changes in the Chinese Pittsburgh Sleep Quality Index (CPSQI) total score from baseline in each group. *indicates a significant difference between the two groups using Mann-Whitney *U*-test (*P* < 0.05). ^a^indicates a significant difference before and after treatment in the experimental group using Wilcoxon signed-rank test (*P* < 0.05).

Changes in the Chinese Pittsburgh Sleep Quality Index (CPSQI) scale of study subjects are shown in Figure 3. The result indicates that statistically significant decreases in the total score before and after treatment were observed in the experimental group (*P* < 0.001). However, in the control group, no significant difference was observed across the same time period (*P* = 0.776). When the Mann-Whitney *U*-test was used to compare the effect of change in the CPSQI total score before treatment between both groups, the data showed a statistically significant difference between the experimental and control groups (−4.90 versus −0.26, *P* < 0.001).

## 4. Discussion

We found a significant decrease in mean HR and increases in time domain analysis HRV parameters (SDNN, RMSDD) after 20 min of lavender inhalation. Moreover, HF is also increased, and all these indices have been used to reflect primarily parasympathetic influences [17, 18]. LF and LFnu remain unchanged. As a result, the LFnu to HFnu ratio is unchanged. LF has been shown to reflect both sympathetic and parasympathetic influences, making the contributive components of this measurement less clear [17, 18]. However, no change in LF and ratio of LFnu/HFnu infers that there is no impact on sympathetic drive to the heart. Our data demonstrated that lavender inhalation may have a persistent short-term effect on HRV with an increase in the vagal tonic and a preserved sympathetic tone. We also showed that 24 sessions of aromatherapy improve sleep quality in women with insomnia up to 1 week after the end of the intervention. The beneficial effect of HRV, however, is not shown in the resting measurements after 4 and 12 weeks of lavender aromatherapy in comparison with the control group.

Prior investigations on the responses of ANS to olfactory stimuli focused on the immediate correlation with subjective mood changes [11, 19]. Saeki investigated the effect of footbath with or without the essential oil of lavender on the ANS of young women [20]. Using spectral analysis, the parasympathetic nerve activity increased significantly during both types of footbath. With the addition of essential oil of lavender, there were positive effects on HRV 10 min after the footbath was complete [20]. Bradley et al. demonstrated that after oral administration of 200 *μ*L lavender essential oil, the amount of linalool was detectable in the blood stream within 10 min and reached a peak at about 30 min and was no longer detectable by 45 min [21]. Our result confirmed the above observation of the consistently short-term effect of lavender inhalation on the modulation of HRV. It has been suggested from animal studies that lavender's anxiolytic effects may become more potent with chronic administration (2 weeks) in female rats similar to that of the anxiolytic diazepam [22]. Our data did not support the long-term effect of lavender on the modulation of HRV; thus, the hypothesis that chronic application of lavender aromatherapy would improve autonomic modulations could not be proved.

It has been suggested that insomnia is associated with inappropriate physiological arousal [23]. Patients with primary insomnia have been found to have increased high-frequency EEG activation, abnormal hormone secretion, increased whole body and brain metabolic activation, and elevated HR and sympathetic nervous system activation during sleep [23]. The physiological hyperarousal has been evidenced by increased LF and decreased HF across all sleep stages; both measures reflect elevated sympathetic nervous system activity [24, 25]. A reduced nocturnal HRV as indicated by a lower wake-to-sleep HR reduction and lower SDNN has been shown in a previous report on polysomnographically determined insomnia patients [26]. Additionally, reduced parasympathetic activity as indicated by decreased HF of HRV, as well as decreased RMSSD, was also demonstrated in insomnia patients with objectively determined short sleep duration [26]. We demonstrated that lavender aromatherapy decreases HR and increases HF, SDNN, and RMSSD during a period of 30 min. It might suggest that increases in HRV are an indication that lavender interacted with the parasympathetic nervous system to modulate anxiety and aid in improving self-reported sleep quality. It has been shown that “falling asleep” is preceded by changes in ANS signaling, notably an increase in parasympathetic tone and reduction of sympathetic drive with a concomitant reduction in the HR and an increase in HRV [24, 25, 27]. These changes are thought to be linked to the restorative functions of the parasympathetic nervous system and may be instrumental in the subjective experience of sleep as refreshing [27]. However, it remains unclear whether the change in sympathovagal balance reflects an influence of sleep mechanisms over the ANS, or the reverse [25].

On the basis of a comprehensive review, Herz concluded that the perceived quality of the odor was the most relevant factor accounting for individual responses to aromatherapy [11]. Cognitive or psychological mechanisms of odor transduction may confound pharmacological effects of aromatherapy in humans [19, 28]. Recently developed neuroimaging techniques provide new insights into the role of brain in correlation with autonomic modulation [29, 30]. In a study involving a combination of continuous electrocardiographic (ECG) monitoring and positron emission tomography (PET) examination, Duan and colleagues tried to detect changes of ANS and localization of cerebral activity during lavender aromatic immersion [30]. They found increases in the parasympathetic tone after the lavender fragrance stimulus increases in the HF component and decreases in the LF/HF. Simultaneous measurement with positron emission tomography (PET) suggested that lavender aromatic treatment induced not only relaxation but also increased arousal level in these subjects [30]. Whether the effect of increasing HRV from lavender aromatherapy is through the psychological process or direct pharmacological effect could not be determined from this study. The lack of chronic effect on the modulation of HRV as shown from our data indicates that further investigation needs to be conducted on the interaction of psychological with physiological mechanisms.

The strengths of this study involved consecutive participants with a longitudinal followup. We applied a standardized procedure to deliver the lavender odor and recruited sufficient and representative participant populations to be tested. However, our study also has several limitations. First, polysomnography was not included to monitor specific sleep stages. The recorded differences in HRV data could not be related to specific sleep stages and were rather related to general sleep quality. Second, the participants of this study were a mixture of premenopausal and postmenopausal women with different hormone status. Although prior findings are not entirely consistent, low endogenous estradiol states have been associated with reduced cardiac vagal control [31, 32]. Reproductive hormones were not measured in this investigation; therefore, their role cannot be determined here. Finally, there is no placebo effect in the control group. It appears that participant expectations regarding likely efficacy may have impacts on the outcomes of aromatherapy that have been reported [28]. To minimize this bias, we provided a health education program regarding good sleep hygiene in both groups. Those with no intervention in the control group were offered the same intervention procedure if they wanted. However, further research on the normal sleepers in combination with appropriate control needs to be conducted to clarify the efficacy of lavender aromatherapy in the management of insomnia or other health issues.

## 5. Conclusions

After 12 weeks of lavender aromatherapy, midlife women with insomnia have improvement in the quality of their sleep. Increased HRV modulations with a lower resting HR as well as a higher SDNN, RMSDD, and HF were observed shortly after lavender inhalation. Lavender aromatherapy does not appear to confer benefit on HRV in the long-term followup.

## Figures and Tables

**Table 1:** Demographics and characteristics of study subject between experimental and control groups (*N* = 67).

Variable	Control group (*n* = 33)	Experimental group (*n* = 34)	*P* value
Age (years)^1^	51.09 ± 3.73	50.85 ± 3.73	0.796
Education, *n* (%)^2^			0.777
Below junior	6 (19.4)	5 (15.2)	
High school	18 (58.1)	22 (66.7)	
Above university	7 (22.6)	6 (18.2)	
Employment, *n* (%)^2^			0.941
No	9 (27.3)	9 (26.5)	
Yes	24 (72.7)	25 (73.5)	
Marital status, *n* (%)^2^			1.000
Unmarried	2 (6.1)	3 (9.1)	
Married	27 (81.8)	26 (78.8)	
Divorce	3 (9.1)	2 (6.1)	
Widow	1 (3.0)	2 (6.1)	
Menopause, *n* (%)^2^			0.901
No	16 (48.5)	17 (50.0)	
Yes	17 (51.5)	17 (50.0)	
Regular exercise, *n* (%)^2^			0.675
No	11 (34.4)	13 (39.4)	
Yes	21 (65.6)	20 (60.6)	
Afternoon nap, *n* (%)^2^			0.938
No	21 (65.6)	22 (64.7)	
Yes	11 (34.4)	12 (35.3)	
Drinks coffee/tea, *n* (%)^2^			0.805
No	15 (45.5)	16 (48.5)	
Yes	18 (54.5)	17 (51.5)	
Drinks wine, *n* (%)^2^			0.183
No	28 (75.8)	30 (88.2)	
Yes	5 (24.2)	4 (11.8)	
Smoking, *n* (%)^2^			0.672
No	31 (93.9)	29 (87.9)	
Yes	2 (6.1)	4 (12.1)	
CPSQI-total^3^	9 (8, 12)	11 (9, 13)	0.100

Values are expressed as ^1^mean ± standard deviation; ^2^number (percentage), and ^3^median (interquartile range). *P* Values are based on ^1^independent two-sample *t*-test, ^2^Chi-square test and ^3^Mann-Whitney *U*-test.

**Table 2:** Comparison of HRV parameters in the 4th and 12th weeks before and after lavender fragrance stimuli in the experimental group.

Variable	Before inhalation	After inhalation	*P* value
HR (bpm)			
4th week	72.5 (65, 77)	68 (64, 72)	<0.00*
12th week	77 (73, 88)	74 (67, 82)	<0.00*
SDNN (ms)			
4th week	34.25 (22.84, 45.99)	35.32 (30.07, 46.80)	0.007*
12th week	23.60 (20.39, 39.07)	34.52 (22.74, 40.95)	0.011*
RMSSD (ms)			
4th week	24.58 (15.97, 36.75)	28.68 (23.27, 38.93)	0.003*
12th week	18.26 (14.52, 27.29)	23.25 (19.06, 35.33)	0.031*
TP (ms^2^)			
4th week	804.5 (461, 1443)	887.5 (610, 1896)	0.132
12th week	497 (236, 1211)	781 (310, 1228)	0.299
VLF (ms^2^)			
4th week	279.88 (179.63, 654.97)	418.41 (206.44, 842.06)	0.228
12th week	224.23 (106.74, 461.38)	318.36 (121.96, 489.44)	0.468
LF (ms^2^)			
4th week	213.74 (94.89, 499.63)	284.64 (102.17, 557.60)	0.602
12th week	114.02 (53.36, 454.59)	202.22 (63.92, 344.73)	0.652
HF (ms^2^)			
4th week	140.66 (74.88, 270.39)	183.56 (104.89, 341.60)	0.039*
12th week	102.42 (48.33, 183.01)	153.12 (95.31, 236.38)	0.036*
LFnu (%)			
4th week	61.37 (39.65, 75.22)	62.17 (41.15, 75.46)	0.663
12th week	62.75 (52.63, 66.82)	50.54 (39.02, 75.26)	0.065
HFnu (%)			
4th week	38.63 (24.78, 60.35)	37.83 (24.54, 58.85)	0.663
12th week	37.26 (33.18, 47.37)	49.46 (24.74, 60.98)	0.065
LFnu/HEnu (ratio)			
4th week	1.59 (0.66, 3.04)	1.65 (0.70, 3.07)	0.912
12th week	1.68 (1.11, 2.01)	1.02 (0.64, 3.04)	0.134

Values are expressed as median (interquartile range). *indicates a significant difference before and after treatment using Wilcoxon signed-rank test (*P* < 0.05).
